# Book Review: Tortillas Wheat Flour and Corn Products

**DOI:** 10.3389/fnut.2016.00034

**Published:** 2016-08-18

**Authors:** Silverio García-Lara

**Affiliations:** ^1^Center of Biotechnology, Tecnologico de Monterrey, Monterrey, Nuevo Leon, Mexico

**Keywords:** tortillas, wheat flour, corn, lime-cooked, snacks

Corn, *Zea mays* L., is the cereal with the highest production rate around the world, with an estimated production that exceeded 1021 million tons in 2014 (1). Corn is also the staple food for over 900 million people located in sub-Saharan Africa, South Asia, and Latin America. Around 600 million people are corn dependent, including 120 million malnourished children (2). As an example, the average per capita consumption of corn in Mexico is more than 125 kg/year (342 g/day), and it provides one-third of the total daily calorie intake (1).

Likewise, wheat, *Triticum aestivum*, is the world’s most relevant food crop and the most traded. In 2014, global production of wheat reached 728 millions of tons (1). In the developing world, it is the second most important crop after rice. Wheat feeds about 2.5 billion of poor people in 90 countries (3). Wheat provides 20% of the total calories consumed globally and is considered also as a crucial source of protein. Approximately 80 million farmers in the developing world depend on wheat for their livelihoods (2).

Corn and wheat are the base for the manufacturing of an array of important staples, including tortillas, which are considered as the most important staple food for the Mexico and some Latin American countries (1). The relevance of both corn and wheat tortillas are beyond Latin American countries. Nowadays, it is possible to find tortilla products in every continent around the world thanks to the global commercialization of foods. Corn tortillas and related products are obtained from two main manufacturing processes that include traditional and commercial dry-masa flours (4). Taking all these aspects into account, this book aimed to: (a) provide a deep review of production and processing of tortillas for both corn and wheat, (b) the nutritional and nutraceutical features related to these important food products, and (c) provide full technical information for understanding the properties, quality assurance, and the industry involved in the making and different uses of tortillas.

The book deserves credit for being the first of its kind in the market. Furthermore, the specific attraction of this volume is its comprehensive and outstanding collection of the current scientific and technical information about the history, nutritional/nutraceutical properties, industrial processing and manufacturing, end-quality control, and technology of tortillas and their related products. Needless to say, the tortilla industry and academia needed this comprehensive an updated reference book. In my opinion, the editors and authors have done an excellent job bringing all this knowledge together as a unique compendium.

The book comprises 13 chapters wrote by 11 authors but compiled for 2 of them and takes the readers through an exciting journey of history and the wide array of industrial processes used to manufacture tortillas in a stepwise, reader-friendly style. The structure of each chapter is compact, and its sequential arrangements are an additional benefit to the readers. Most of the chapters are very well focused and have succeeded to introduce their topics and highlighted the state-of-the-art aspects for each specific topic. The content is written clearly and employs a comprehensive English language.

This book starts with a general review of history of corn and wheat tortilla then, a description to the nutrition, phytochemical and nutraceutical properties of tortillas, and a discussion of how these properties could be enhanced by fortification and supplementation with flours and additives. One of the major trends in the food industry is to develop new food products with added value (5). Effects of types of flours and the methods for quality assurance (production and food safety) on tortilla manufacturing are considered in separate chapters (4, 5, and 6). Three chapters then deal with different aspects of development and quality end-product (leavening, dough conditioners, preservatives, stabilizers, and enzymes), and the effects on shelf stability and eating quality of tortilla. The processing of flour tortillas is also discussed, particularly traditional processing, industrial processing, and differences in tortilla properties (Chapter 5). The next chapter describes the main flour tortilla problems, distinguishing the defects, and possible solutions for each problem. The final two chapters consider different aspects of food grade corn quality for lime-cooked products and industrial production of tortillas and snacks, either as review of the key ingredients or of massive production using alternative processing technologies. The book contains examples from many different technologies, procedures, derived products, and the interactions between corn, wheat, and others crops and legumes for new tortilla products.

Inside the book, clear diagrams and models are absolutely essential for understanding basic and advanced concepts of tortilla processing, and relevant figures make the volume more attractive. Outstanding illustrations, rich tables, flow charts, and diagrams allow the reader to easily understand the contents and are especially helpful for no experienced students.

However, one of the critics for this book has been that, unfortunately, recent literature on the subject has not been included in some of the chapters. In addition, two of the chapters are not well organized in terms of figures and color pictures, which could cause a disturbance in the reading flow. Advanced readers would appreciate more emphasis on global trends of tortilla industry and recent advances in barley, sorghum, and other raw material to produce new tortilla products.

In summary, this new book is an excellent resource on central aspects of wheat flour and corn tortilla products. It will quite effectively meet the needs for academics and industry professionals, including food science, food agro-technologist, and nutrition students and people working in the tortilla and snack food industries including quality control/assurance and food product developers. Finally, it is worthwhile to mention that this book was dedicated to Dr. Ralph Waniska, who conceptualized the original idea of writing the title and spent most of his life researching the basic and practical aspects of tortillas. Today, people in academia and industry still benefit for his contributions.

This book can be found at: http://www.aaccnet.org/publications/store/pages/27885.aspx

## Author Contributions

SGL wrote and reviewed the latest version of this manuscript.

## Conflict of Interest Statement

The author declares that the research was conducted in the absence of any commercial or financial relationships that could be construed as a potential conflict of interest.
